# Optimal Community Assembly Related to Leaf Economic- Hydraulic-Anatomical Traits

**DOI:** 10.3389/fpls.2020.00341

**Published:** 2020-03-25

**Authors:** Congcong Liu, Ying Li, Jiahui Zhang, Alec S. Baird, Nianpeng He

**Affiliations:** ^1^Key Laboratory of Ecosystem Network Observation and Modeling, Institute of Geographic Sciences and Natural Resources Research, Chinese Academy of Sciences, Beijing, China; ^2^College of Resources and Environment, University of Chinese Academy of Sciences, Beijing, China; ^3^The Key Laboratory for Forest Resources & Ecosystem Processes of Beijing, Beijing Forestry University, Beijing, China; ^4^Department of Ecology and Evolutionary Biology, University of California, Los Angeles, Los Angeles, CA, United States; ^5^Institute of Grassland Science, Northeast Normal University, and Key Laboratory of Vegetation Ecology, Ministry of Education, Changchun, China

**Keywords:** functional traits, ecosystem productivity, forest, community composition, community coexistence, trait moment, leaf function

## Abstract

Multi-dimensional trait mechanisms underlying community assembly at regional scales are largely unclear. In this study, we measured leaf economic, hydraulic and anatomical traits of 394 tree species from tropical to cold temperate forests, from which we calculated the leaf trait moments (mean, variance, skewness, and kurtosis) using community-weighted methods. Economic and hydraulic traits were decoupled at the species level, but coupled at the community level, and relationships between leaf traits in observed communities were stronger than that in null communities, suggesting that the adaptive mechanisms of plant species may be different. Furthermore, leaf economic traits were distributed more evenly across species occupying communities with lower temperature and precipitation, whereas hydraulic traits were distributed more evenly under lower water availability. This suggests that limiting similarity of specific leaf traits within communities would be enhanced when related-resources are limited, and highlights the independent assembly of leaf economics and hydraulic traits in terms of functional evenness. Importantly, the moments of leaf economic and hydraulic traits of observed communities explained more variation in ecosystem productivity than that of null communities, indicating ecosystem productivity depended on trait-based community assembly. Our results highlight the principles of community assembly regarding multi-dimensionsional traits in natural forests at a regional scale.

## Introduction

How plant communities assemble and persist has been a fundamental question of ecology (Götzenberger et al., 2012), and trait-based approaches have recently been proposed as an effective way to reveal the underlying principles (Mcgill et al., 2006; Westoby and Wright, 2006; Cadotte, 2011). Two important processes play crucial roles in community assembly: habitat filtering and limiting similarity (Diaz et al., 1998; Weiher et al., 1998). Previous investigation of non-random assembly of plant traits with respect to communities theoretically and empirically demonstrated that limiting similarity may result in ecological differentiation of coexisting species and habitat filtering reduces the range of trait values (Cornwell et al., 2006; Cornwell and Ackerly, 2009; Bernardverdier et al., 2012). These studies expanded our understanding of trait-based community assembly, however, it is still unclear how multi-dimensional traits (such as leaf economic, hydraulic, and anatomical traits) and their interaction determine community assembly processes.

Relationships between pairs of leaf traits (hereafter called trait-trait relationships) at the species level are well-explored, but can arise for varying reasons (Sack and Scoffoni, 2013). First, some trait-trait relationships are directly mechanistic (i.e., physiological structure function relationships), where for instance the size or number of a given structure determines the physiological output of a process. Second, some arise due to their being co-selected due to optimal design, i.e., each trait independently contributes structurally to an overarching function. In addition, certain leaf hydraulic traits, such as stomatal traits, are closely related to water-use and leaf temperature maintenance and are typically correlated to venation traits to maintain water balance (Sack et al., 2003; Sack and Frole, 2006; Li et al., 2015). Lastly, some arise due to concerted convergence of the two traits, i.e., each trait contributes independently to an advantage in the given environment. Despite these varying mechanisms underlying trait-trait relations, certain relationships between leaf traits are of exceptional importance given their consistent slopes across species among vastly different biomes (Reich et al., 1999). While some studies have investigated the coordination between leaf economics and hydraulic traits at the species level (Li et al., 2015; Yin et al., 2018), little knowledge concerning whether these two sets of traits vary in a fully coordinated manner or whether independent variation would decouple such relations at the community level.

Assembly of different plant species within communities could alter the relationships between economic and hydraulic traits at the community level. Accordingly, trait-trait relationships established across species of varying communities could potentially be different than trait-trait relationships at the community level (Bruelheide et al., 2018). Indeed, leaf economic and hydraulic traits were decoupled across species from tropical and subtropical forests (Li et al., 2015). By contrast, coupling of such traits in low resource communities would provide greater efficiency with respect to carbon cost (Flores-Moreno et al., 2019) and could help plants adapt to drought stressed environments (Yin et al., 2018). Here, we assume that the coupling of leaf economic and hydraulic traits through filtering specific plant species might be an adaptation strategy of plant communities.

The niche-based theory of community assembly proposes that niche differentiation is the basis of species coexistence, and each species can occupy a unique niche within a stable community in the long run (Macarthur and Levins, 1967). When essential resources are limited in a given community, competition occurs between different species, and finally the process of limiting similarity may reduce the overlap of plant species and lead to an even spacing of species along the specific resource axes and platykurtic distribution within communities (Stubbs and Wilson, 2004). For example, leaf hydraulic traits (e.g., maximum stomatal conductance) have a close linkage with water use efficiency and can reflect the water use strategies of plants (Franks et al., 2015; Liu et al., 2018). Zhang et al. (2017) found that divergences in hydraulic traits formed an important basis for niche differentiation. Within a shrub-steppe community, hydrologic niches explained species coexistence and abundance, and despite high overlap in trait values across species, small differences in hydrologic niches are fundamentally important (Kulmatiski et al., 2019). The latter suggests that plants with slightly different water use strategies within communities could make water use efficiency optimal under drought stress environments. Whether or not leaf hydraulic traits are distributed more evenly within plant communities under limited water supply has not been tested.

Traits involved in the physiological functioning of leaves are fundamental in determining species growth capacities in their given environments, and by extension the productive capacity of the ecosystem. Indeed, plant traits have a close linkage with ecosystem productivity, and ecologists are eager to incorporate plant traits into ecological models to improve their predictions (Croft et al., 2017; Luo et al., 2019). For example, stomatal density can account for 51% of the variation in ecosystem productivity (Wang et al., 2015). Chlorophyll content (Li et al., 2018), palisade tissue thickness (He et al., 2018), and leaf nitrogen and phosphorus content (Tang et al., 2018) also have important influences on forest ecosystem productivity. However, most of these studies only focused on the community-weighted means of functional traits. Gross et al. (2017) and Zhang et al. (2019) reported that variance, skewness, and kurtosis of traits were strongly correlated with ecosystem functions in drylands and grasslands, respectively. Thus, whether and how trait moments influence ecosystem productivity across forests remains unclear.

In this study, we explored the principles of community assembly in natural forests using multi-dimensional trait analyses. Specifically, we tested two hypotheses: (1) economic-hydraulic traits are strongly coordinated at the community level but decoupled at the species level, and (2) leaf hydraulic traits are distributed more evenly under environments with lower water availability. We then explored whether and how trait-based community assembly determines ecosystem productivity. To test the above hypotheses and answer the aforementioned uncertainties, we measured nine leaf functional traits, including leaf economic, hydraulic and anatomical traits of 394 species of tropical to cold temperate forests.

## Materials and Methods

### Study Sites

**FIGURE 1:**
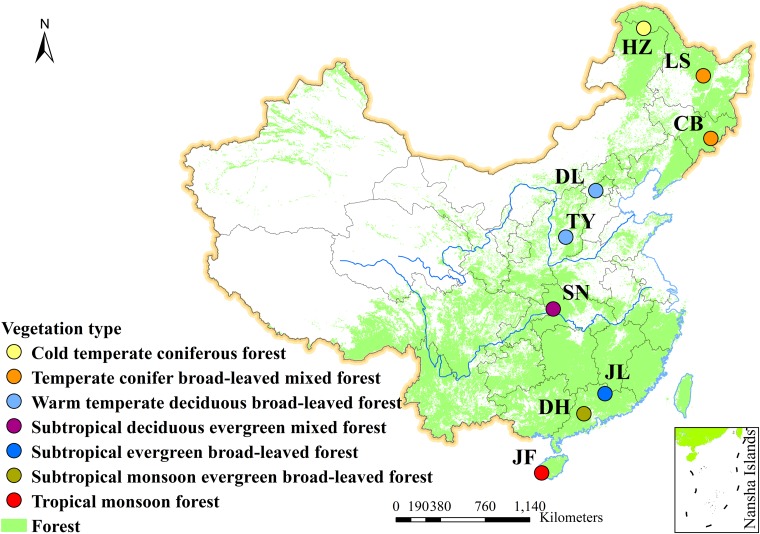
Geographic distribution of sampling sites along the North-South Transect of Eastern China (NSTEC). CB, Changbai; DH, Dinghu; DL, Dongling; HZ, Huzhong; JF, Jianfeng; JL, Jiulian; LS, Liangshui; SN, Shennong; TY, Taiyue. Site descriptions are shown in Supplementary Table S1.

Nine typical forests were selected along the 3700-km north-south transect of China (NSTEC), and they were designated as Jianfengling (JF), Dinghu Mountain (DH), Jiulian Mountain (JL), Shennongjia (SN), Taiyue Mountain (TY), Dongling Mountain (DL), Changbai Mountain (CB), Liangshui (LS), and Huzhong (HZ) (Figure 1). These forests extend from 18.7 to 51.8°N latitudes, and represent most of the forest types in the Northern hemisphere (He et al., 2019), including cold-temperate coniferous, temperate deciduous, subtropical evergreen, and tropical rain forests (Supplementary Table S1). Within each forest, our measurements were conducted from four communities, resulting in 36 total communities.

### Sample Collection

The field survey was conducted from July–August 2013, the peak period of growth for all forests. Sampling plots were located within well-protected national nature reserves with relatively continuous vegetation that is representative of the given forest types. Four experimental plots (30 m × 40 m) were first established in each forest. Plant height and diameter at breast height of all individuals within plots were measured to estimate leaf biomass (see Materials and Methods section “Leaf trait moments of plant communities”). In addition to collecting tree species within the sampled community, tree species around the community were also collected, which covered almost all the tree species that could inhabit the sampled community. Sampling outside of communities allowed us to assess for differential trait distributions that may arise within versus across communities.

For each tree species, mature sun exposed leaves (*n* = 20–30) were collected from four healthy trees and mixed as a composite sample, and then the leaf samples were divided equally into three parts each with approximately 10 leaves. Leaves from one part were used to measure leaf size, dry weight and nitrogen concentration, from another were immediately stored in a cool box with ice and then into the refrigerator to measure chlorophyll concentration, and the last part were cut into small pieces (1.0 cm × 0.5 cm) along the main vein and fixed in formalin-acetic acid-alcohol (FAA; 5 ml of 38% formalin, 5 ml of glacial acetic acid, 90 ml of 50% ethanol, and 5 ml of glycerin) to measure hydraulic and anatomical traits (Chen and Wang, 2009).

### Measurement of Leaf Economic-Hydraulic-Anatomical Traits

Two leaf economic traits were measured, including specific leaf area (SLA) and leaf nitrogen content (N), and we additionally determined leaf chlorophyll content (Chl). After sampling, leaf size was measured using a scanner (Cano Scan LIDE 100, Japan) and Photoshop CS software (Adobe, United States), then these leaves were subsequently dried to constant weight in an oven to measure leaf dry weight, SLA was the ratio of leaf size to leaf dry weight (Wang et al., 2016; Liu et al., 2019). All dried leaf samples were ground to fine powder using an agate mortar grinder (RM200, Retsch, Haan, Germany) for elemental analysis (Zhang et al., 2018), and leaf N concentration was measured using an elemental analyzer (VarioMAX CN Elemental Analyzer, Elementar, Hanau, Germany). Fresh leaves were cleaned to remove soil and other contaminants, and 0.1 g of fresh leaves was used to extract chlorophyll using 95% ethanol, with four replicates for each species, and leaf chlorophyll concentration (Chl) of the filtered solution was measured using the classical spectrophotometric method with a spectrophotometer (Pharma Spec, UV-1700, Shimadzu, Japan) (Li et al., 2018).

Stomatal and venation traits mechanistically influence leaf hydraulic function, and these two trait categories are often strongly coordinated with each other (Li et al., 2015; Yin et al., 2018), therefore we took stomatal traits as the proxy of hydraulic traits in this study (Liu et al., 2018). Stomatal traits were observed by scanning electron microscope (S-3400°N, Hitachi, Japan). Three small pieces were selected from the pooled sample, and each replicate was photographed twice on the lower surface (Fixed image area is 1.12 mm^2^). In each image, the number of stomata in each photo was recorded, and stomatal density (*d*) was defined as the number of stomata per leaf area. Five stomata were selected for measurement of stomatal length and width using MIPS (Optical Instrument Co., Ltd., Chongqing, China), and stomatal size (*s*) was the area of an ellipse calculated from dimensions of major axis complex length and the minor axis complex width, including the pore size within. Stomatal area fraction (*f*) was the proportion of total stomatal area, calculated as the product of stomatal density and size, to leaf area.

Leaf anatomical traits play important roles in gas exchange and water transport (Sack and Frole, 2006; Yin et al., 2018). Spatial anatomical separation between leaf economic and hydraulic traits has been found across species, where leaf economic traits, such as nitrogen content were mainly attributed to the palisade mesophyll while hydraulic traits were mainly linked with spongy mesophyll (Li et al., 2015), therefore palisade tissue thickness (PT) and spongy tissue thickness (ST) were measured. Three small pieces selected from the pooled sample were progressively dehydrated in an ethanol series (50%, 70%, 85%, 95%, and 100%) and infiltrated with warm paraffin; leaf transverse sections (8–12 μm) thickness were then cut using a rotary microtome (Leica RM2255, Germany). The slides were stained using safranin and fast green (1% aqueous safranin and 0.5% fast green in 95% ethanol), then PT and ST were measured using an electronic image analysis equipment (MIPS software, Optical Instrument Co., Ltd., Chongqing, China), each small pieces was photographed twice, the PT and ST were measured 5 times in each photo. In total, each anatomical trait was measured 30 times for each plant species (He et al., 2018). We then calculated the ratio of PT to ST (PT/ST).

Abbreviations, units of nine leaf functional traits, and trait profiles are summarized in Table 1.

**TABLE 1:** Variation in 9 leaf functional traits measured for 394 tree species along the North-South Transect of Eastern China (NSTEC).

Leaf functional traits		Abbr.	Unit	Mean	SE^†^	CV	Skew	Kurt	Max	Min
Economic	Specific leaf area	SLA	m^2^ kg^–1^	12.52	0.37	0.58	1.47	2.94	46.76	1.89
	Leaf nitrogen content	N	%	2.16	0.04	0.35	0.98	1.25	5.32	0.73
	Leaf chlorophyll content	Chl	mg g^–1^	5.7	0.12	0.4	0.85	2.13	17	0.93
Hydraulic	Stomatal density	*d*	#mm^–2^	253.26	9.16	0.72	1.63	4.17	1128	1.49
	Stomatal size	*s*	μm^–2^	379.52	13.32	0.7	2.47	9.17	2198.2	33.3
	Stomatal area fraction	*f*	%	8.39	0.34	0.8	1.66	3.46	38.93	0.08
Anatomical	Palisade tissue thickness	PT	μm	27.65	0.68	0.49	1.45	4.7	113.97	3.12
	Spongy tissue thickness	ST	μm	63.57	2.05	0.64	1.69	3.82	257.41	8.03
	Ratio of PT to ST	PT/ST	%	53.52	1.51	0.56	1.66	4	192.25	8.45

*^†^SE, standard error (n = 394); CV, coefficient of variance.*

### Leaf Trait Moments of Plant Communities

Total leaf biomass of each individual was calculated using species-specific allometric regressions based on measured values of diameter at breast height and tree height, and then leaf biomass of each species within plots were calculated. These equations were obtained from the Chinese Ecosystem Research Net database (He et al., 2019), including published and some unpublished data (Supplementary Table S2). Allometric relationships might vary with climatic conditions and with species, so it is virtually unrealistic to exactly match so many plant species with its allometric equation. Therefore, we used the biomass equations from the same genera, or mixed-species equations of a forest to estimate leaf biomass when their allometric equations were unavailable. In total, 246 species-specific allometric equations (R^2^ ranged from 0.52 to 1) were used in this study.

The mass ratio hypothesis (Grime, 1998; Garnier et al., 2004) predicts that the extent to which the trait of a given species affects ecosystem properties depends on the relative contribution of that species to the total community biomass. Accordingly, the community-weighted mean trait values (Mean) can better reflect the local ‘optimal’ trait strategy, for which we calculated for a given trait as:

(1) $$M\,e\,a\,n=\sum_{i}^{n}p_{i}\,T\,r\,a\,i\,t_{i}$$

where n was the number of species sampled in the plant community, *p*_*i*_ was the relative leaf biomass of *i*^*th*^ species, and Trait*_*i*_* was the specific leaf functional trait of the *i*^*th*^ species.

However, community-weighted mean has its inherent flaws, for instance by potentially mistakenly exacerbating the role of dominant species and by masking the other important information of trait distributions within the community (Bagousse-Pinguet et al., 2017). Therefore, in this study, we not only focus on the community-weighted mean of traits, but also community-weighted variance (Variance), community-weighted skewness (Skewness), and community-weighted kurtosis (Kurtosis), which were calculated as follows:

(2) $$V\,a\,r\,i\,a\,n\,c\,e=\sum_{1}^{n}p_{i}\,{\left(T\,r\,a\,i\,t_{i}-M\,e\,a\,n\right)}^{2}$$

(3) $$\text{Skewness}=\sum_{1}^{n}{\frac{p_{i}\,\left(T\,r\,a\,i\,t_{i}-M\,e\,a\,n\right)}{V\,a\,r\,i\,a\,n\,c\,e^{3/2}}}^{3}$$

(4) $$\text{Kurtosis}=\sum_{1}^{n}{\frac{p_{i}\,\left({\text{Trait}}_{i}-\text{Mean}\right)}{{\text{Variance}}^{2}}}^{4}$$

As such, the variance signifies the amount of scale or dispersion of the trait value distribution in a community. Skewness signifies the level of left or right tailed asymmetry of the distribution of trait values, and highly absolute skewness means dominant traits locate in the extreme of the trait–abundance distribution. Such outcomes typically result from asymmetrical competition and/or rapid environmental change such as disturbance. Kurtosis signifies the relative extremes throughout the trait distribution, where higher values result from a community having species of high abundance with similar trait values (i.e., low diversity) and lower values results from a community with species of even abundance but differential trait values (i.e., high diversity). Indeed, mean and skewness of traits within communities reflect the patterns of trait dominance, and highly absolute skewness reflected high asymmetry of the trait distribution and is often associated with hierarchical competition. Variance and kurtosis of traits within communities could reflect two components of functional diversity: dispersion and evenness. Skewness and Kurtosis are mathematically related and their relationship is described as:

(5) $$\text{Kurtosis}\geq {\text{Skewness}}^{2}+1$$

a full mathematical demonstration of the Skewness- Kurtosis relationship, and its implications and use in various scientific fields were shown in Supplementary Note 1 of Gross et al. (2017).

Thus, a particular value of skewness of a specific leaf trait within communities must correspond to a lowest kurtosis. Here, the distance (D) between observed kurtosis and the lowest kurtosis (Skewness^2^ +  1) was calculated as:

(6) $$\text{D}=\text{Kurtosis}-\left({\text{Skewness}}^{2}+1\right)$$

where D ranges from 0 to infinity. To obtain approximate normality and homogeneity of residuals in this study, we added one to all the D values before log-transformation (Muir, 2018), and log-transformed D was called δ. Kurtosis of a functional trait could represent functional evenness, and a lower kurtosis reflects high functional diversity (Zhang et al., 2019). The skewness of leaf traits could reflect selection pressures to some extent (Wieczynski et al., 2019). Accordingly, different communities are likely under different strength of selection pressures, so it is not fair to directly compare the kurtosis. Thus, we used this parameter δ to quantify relative kurtosis of trait distribution. Cornwell and Ackerly (2009) pointed out that limiting similarity will affect the spacing and lead to a platykurtic (flat-topped) distribution, so a lower δ value means a relatively more platykurtic distribution and higher functional evenness (even distribution within communities).

### Climate Data and Gross Primary Productivity (GPP)

Climate data from 2000 to 2010 at a spatial resolution of 1 × 1 km were obtained from Resource and Environment Data Cloud Platform^1^. Mean annual precipitation (MAP) and mean annual temperature (MAT) were extracted using latitude and longitude coordinates. The de Martonne aridity index (AI, $\text{AI}=\frac{\text{MAP}}{\mathrm{MAT}+10}$) was used to represent water availability, and a lower AI indicates lower water availability or more severe drought stress (de Martonne, 1926). Notably, soil water availability was not consistent with MAP in this study. Despite MAT and MAP both decreasing with latitude, drought stress in the mid-latitudes was of greater magnitude (Supplementary Figure S1). The GPP data used in this study were retrieved from MODIS (moderate resolution imaging spectroradiometer) data at a 1 × 1 km grid between 2000 and 2010^2^.

### Data Analysis

The mean, variance, skewness, and kurtosis of 36 plant communities for nine leaf functional traits were calculated. Given that the species richness of three plant communities in cold temperate coniferous forest was less than three, the variance, skewness, and kurtosis of these plant communities were not used for further analysis (Gross et al., 2017). Pearson correlations for pairwise combinations of traits were tested, and multiple-trait relationships were analyzed using principal component analysis (PCA, z-transformed) at the species and community levels. We conducted linear regression between climate and δ values, if δ values were significantly influenced by MAT and MAP, then we used their first principal component scores as their proxy (Supplementary Table S3) to plot δ values against climate, given their tight covariance.

Absolute value of the Pearson correlation coefficient was used to describe the strength of the relationship between leaf traits. Coordination between leaf economic and hydraulic traits was tested using the first principal component scores of these two sets of traits (Li et al., 2015), and Pearson correlation coefficient was used to describe their relationship. Partial redundancy analysis (rda function in “vegan” package) was used to explore the influence of leaf trait moments on GPP, and we used GPP as the dependent variable; MAT and MAP as covariates; and the moments of leaf economic traits, hydraulic traits, and anatomical traits as the explanatory variables, respectively.

The null model approach can remove effects of sample size and test the process of community assembly. To create null communities, we took species within forest sites (Null_within, sample size: species richness of focal site) and species across forest sites (Null_across, sample size: 394 plant species) as our species pools. We randomly shuffled trait values using these species pools and null communities were created accordingly (Wang et al., 2018), then relationships between leaf traits, coordination between economic traits and hydraulic traits, the influence of leaf trait moments on GPP (*parameter* in SES equation) were determined. Each null model was performed 9999 times. Previous studies used standardized effect size (SES) to assess non-random patterns of trait distribution (Kraft and Ackerly, 2010), calculated as: $\text{SES}=\frac{{\text{parameter}}_{\text{observed}}-\text{mean}\,\left({\text{parameter}}_{\text{null}}\right)}{\mathrm{standard}\,\mathrm{deviation}\,\left({\text{paramater}}_{\text{null}}\right)}$, community assembly was non-random if SES was significantly different from 0. Here the difference between *parameter*_*observed*_ and *parameter*_*null*_ was directly compared, and if a significant difference was observed, the process of community assembly was non-random.

Data analyses and visualization were performed using R software (version 3.3.1, R Development Core Team 2016). The level of significance was established at *p* < 0.05.

## Results

### Relationships Between Leaf Traits at the Species and Community Level

We observed significant relationships between certain leaf traits and when scaling these traits up to the community level, some trait-trait relationships were slightly different from those at the species level (Table 2). From species to community level, some trait-trait relationships changed from non-correlation to positive or negative correlation, such as stomatal size (*s*) versus palisade tissue thickness (PT), stomatal area fraction (*f*) versus spongy tissue thickness (ST); some trait-trait relationships became stronger, such as nitrogen content (N) versus specific leaf area (SLA), stomatal density (*d*) versus *s*. Lastly, the SLA-PT relationship was completely contrasting between the species and community levels.

**TABLE 2:** Pearson correlation coefficients among 9 leaf functional traits at the species (lower diagonal) and community level (upper diagonal).

	SLA	N	Chl	*d*	*s*	*f*	PT	ST	PT/ST
SLA		**0.76**	**0.81**	0.29	–0.09	0.23	**0.42**	–0.21	**0.56**
N	**0.55**		**0.74**	**0.60**	–0.22	**0.49**	0.09	**−0.36**	**0.57**
Chl	**0.49**	**0.54**		**0.40**	**−0.35**	0.28	0.09	**−0.36**	**0.59**
*d*	–0.02	0.06	0.08		**−0.43**	**0.94**	–0.30	**−0.72**	**0.75**
*s*	**−0.18**	**−0.23**	**−0.23**	**−0.26**		–0.13	**0.37**	0.31	**−0.24**
*f*	–0.10	–0.08	–0.03	**0.63**	**0.38**		–0.26	**−0.73**	**0.76**
PT	**−0.17**	**−0.11**	**−0.16**	0.01	0.01	0.04		**0.62**	–0.06
ST	**−0.31**	**−0.33**	**−0.34**	–0.06	**0.15**	0.05	**0.52**		**−0.79**
PT/ST	**0.24**	**0.31**	**0.26**	**0.12**	**−0.18**	0.01	**0.24**	**−0.53**	

*Significant correlations are indicated in bold. SLA, specific leaf area; Chl, leaf chlorophyll content; N, leaf nitrogen content; d, stomatal density; s, stomatal size; f, stomatal area fraction; PT, palisade tissue thickness; ST, spongy tissue thickness; PT/ST, ratio of PT to ST.*

**FIGURE 2:**
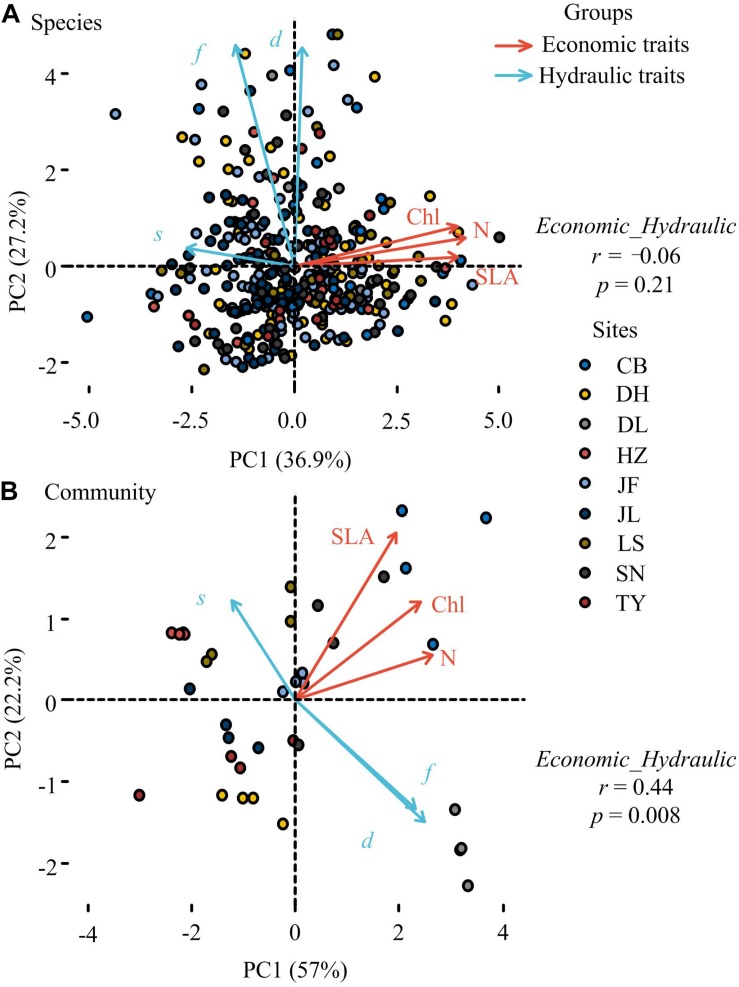
Principal component analysis (PCA) of leaf economic and hydraulic traits at the species **(A)** and community levels **(B)**. SLA, specific leaf area; Chl, leaf chlorophyll content; N, Leaf nitrogen content; *d*, stomatal density; *s*, stomatal size; *f*, stomatal area fraction Points in panel a and panel b represented plant species and plant community, respectively. CB, Changbai; DH, Dinghu; DL, Dongling; HZ, Huzhong; JF, Jianfeng; JL, Jiulian; LS, Liangshui; SN, Shennong; TY, Taiyue. Site description was shown in Supplementary Table S1. Relationships between leaf economic and hydraulic traits were represented by the correlations between the first principal component scores of leaf economic traits and leaf hydraulic traits. We conducted two PCAs for 2 different groups of variables, and the correlations between PC1 scores of leaf economic traits and PC1 scores of leaf hydraulic traits were tested at the species and community levels, respectively.

**FIGURE 3:**
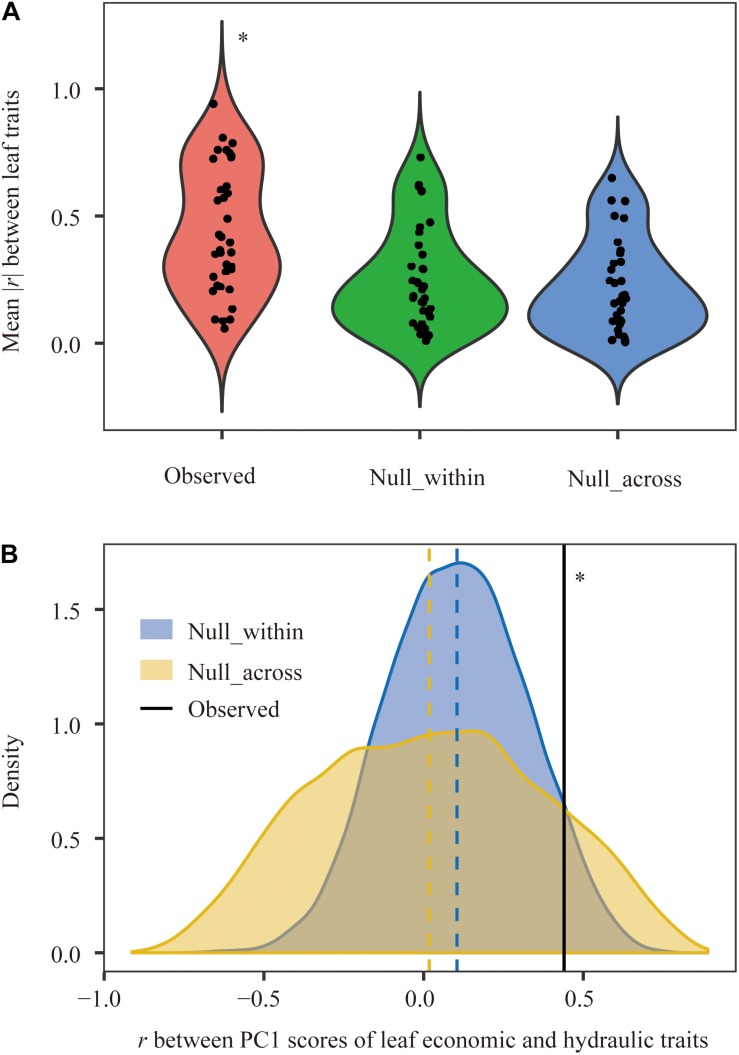
Comparison of correlations among leaf functional traits between observed and null communities at the community level. **(A)** Paired trait-trait correlations among nine leaf traits and **(B)** relationships between leaf economic traits and hydraulic traits. *r*, Pearson correlation coefficient. Vertical dashed line, the mean value for each distribution. ^∗^Parameter in observed plant communities was significantly different from that in null_within and null_across at the 0.05 level.

Principal component analysis showed that leaf economic traits and hydraulic traits were decoupled at the species level but coupled at the community level (Figure 2). Compared with null communities, the trait-trait relationships and the coordination between leaf economic and hydraulic traits in observed communities were much stronger (Figure 3).

### Relationships Between Climate and the Distances of Observed Kurtosis to Boundary Kurtosis (δ)

**FIGURE 4:**
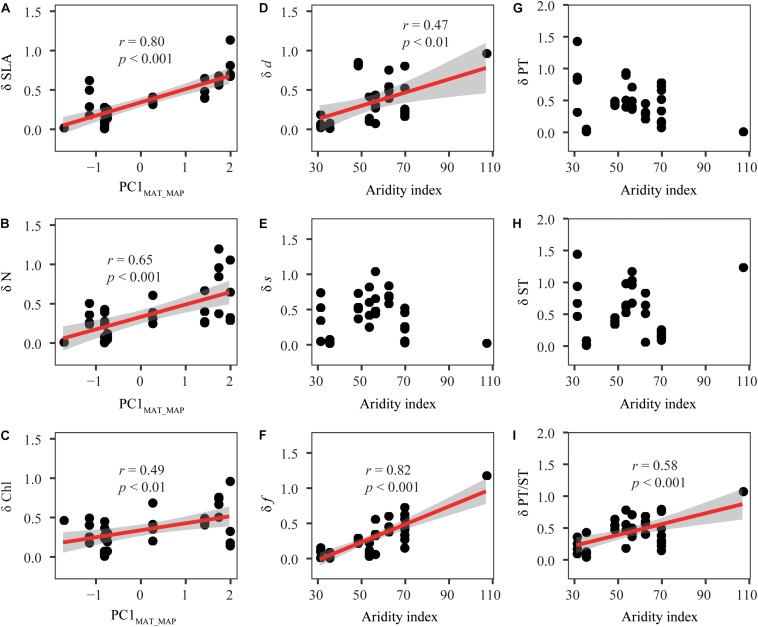
Relationships between the climate variables and the distance (δ) between the observed kurtosis and the boundary kurtosis. **(A–I)** Represent the relationships between the distance (δd) of specific leaf traits and climate. Details were shown in Supplementary Table S3. Each point represents one plant community (species richness ≥ 3). The red lines are fitted using linear regression. Shaded areas indicate the 95% confidence interval. SLA, specific leaf area; Chl, leaf chlorophyll content; N, Leaf nitrogen content; *d*, stomatal density; *s*, stomatal size; *f*, stomatal area fraction; PT, palisade tissue thickness; ST, spongy tissue thickness; PT/ST, the ratio of PT to ST. PC1_*MAT_MAP*_, the first principal component scores of mean annual temperature and precipitation, which accounted for 94.9% of total variation of mean annual temperature and precipitation. δ is the log-transformation of the distance between observed kurtosis and boundary kurtosis. Because some of the original data had very low values close to 0, one was added to all values before log-transformation.

δ values were significantly influenced by mean annual temperature (MAT), mean annual precipitation (MAP) and de Martonne aridity index (AI) (Figure 4 and Supplementary Table S4). Specifically, δ values of SLA, N, and chlorophyll content (Chl) increased with increasing MAT and MAP. δ values of stomatal density (*d*) and stomatal area fraction (*f*) were closely related with AI. δ of palisade tissue (PT) and spongy tissue (ST) were less influenced by climate, but the relationships between δ values of PT/ST and AI were observed.

### The Influence of Leaf Trait Moments on Gross Primary Productivity (GPP)

**FIGURE 5:**
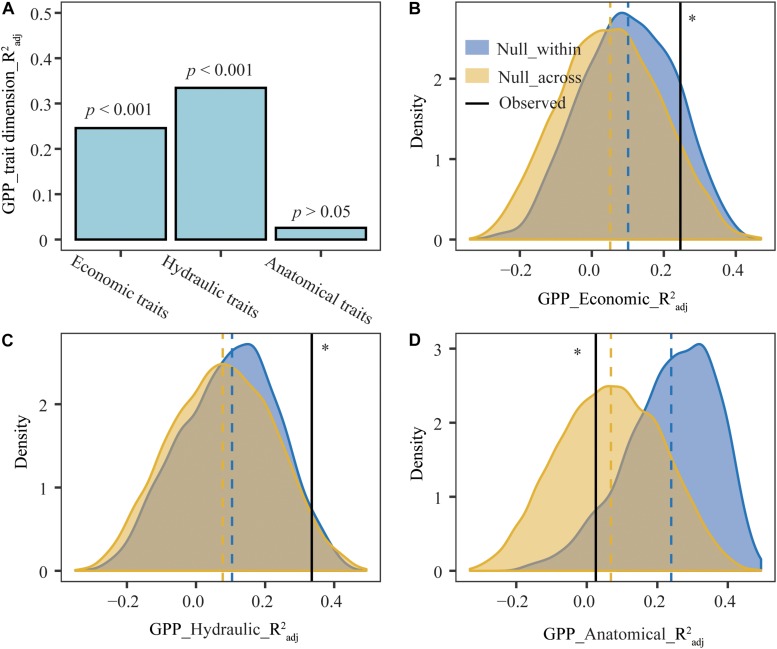
Pure effects of leaf trait moments (mean, variance, skewness, and kurtosis) on gross primary production (GPP) in observed and null plant communities. Vertical dashed line, the mean value for each distribution. **(A)** Observed pure effects of leaf trait moments on GPP. **(B–D)** Distributions of simulative pure effects of leaf trait moments on GPP. GPP_trait dimension_R^2^_*adj*_, the pure effects of single trait dimension on GPP. GPP_Econimics_R^2^_*adj*_, GPP_Hydraulic_R^2^_*adj*_, and GPP_Anatomical_R^2^_*adj*_ represented the pure effects of leaf economic, hydraulic and anatomical traits on GPP, respectively. ^∗^Pure effects of leaf economical, hydraulic, and anatomical traits on GPP in observed plant communities were significantly different from that in null_within and null_across at the 0.05 level.

After including climate variables as covariates, the moments (mean, variance, skewness, and kurtosis) of leaf economic and hydraulic traits accounted for 24.59% and 33.46% of the variation in GPP (Figure 5), however, leaf anatomical traits had no significant influence on GPP. Leaf economic and hydraulic traits of observed communities explained more variation in GPP than that of null communities, while leaf anatomical traits of observed communities explained less variation in GPP than that of null communities.

## Discussion

Multi-dimensional traits—leaf economic, hydraulic and anatomical traits— were used to explore the principles of community assembly from tropical to cold-temperate forests. We found that adaptation mechanisms of plant communities and plant species might be different, evidenced by the decoupling of economic and hydraulic traits at the species level but coupling at the community level. When resources are limited, related leaf traits tended to be relatively even within communities, and trait-based community assembly always optimized their productivity. The insights provided by leaf functional traits in this study help us to better reveal the principles of community assembly in natural forests, and systematic investigation of plant traits (leaf-stem-root functional traits) at a large scale is required for a comprehensive understanding of trait-based community assembly.

### Coordination Across Certain Leaf Traits Scaled From the Species to Community Level

We found a species level trade-off between specific leaf area (SLA) and the palisade tissue thickness (PT) and the spongy tissue thickness (ST) such that more resource acquisitive species had both lower PT and ST. Yet, we also confirmed coordination between SLA and the ratio of PT to ST, suggesting that more resource acquisitive species actually require more PT relative to ST, as this would be consistent with greater resource acquisition (Yin et al., 2018). While the construction of palisade tissue necessitates more carbon investment than spongy tissue (John et al., 2017), the palisade is also much more efficient in terms of light interception and thus photosynthetic capacity, and the trade-off suggests that this would be selected and potentially underlie greater resource acquisition across species. Such pattern was also revealed across communities and suggests that this trade-off is conserved during habitat filtering that drives specific plant species together, irrespective of the climate or species in the given community (Tatarko and Knops, 2018; Song and Liu, 2019). Indeed, these traits were also strongly related to chlorophyll and nitrogen contents at both the species and community levels, reflecting evolution of traits by optimal design, and consistent with greater productivity that would scale to the level of the whole ecosystem (Wright et al., 2004; He et al., 2018). Such relationships highlight the importance of leaf level coordination and physiological function in determining species evolutionary adaptation and assembly into communities of varying climate and species.

### Adaptation Mechanisms of Plant Communities and Plant Species Might Be Different

We found decoupling of leaf economic and hydraulic traits at the species level across 394 species from tropical to cold temperate forests, as has been demonstrated across 85 woody species in tropical-subtropical forests (Li et al., 2015). A lack of coupling between these traits is consistent with their contrasting impacts on leaf level function (Li et al., 2015). Indeed, decoupled relationships between leaf economic and hydraulic traits potentially allow greater evolvability for more combinations of leaf traits to adapt to multifarious niche dimensions, resulting in many plant species that coexist within communities in tropical-subtropical forests (Li et al., 2015; Flores-Moreno et al., 2019).

Trait-trait relationships represent coordinated ecological strategies and trade-offs of plants, due to the different strategies that could facilitate co-existence (Bruelheide et al., 2018). Yet, such relationships may not be the same at the species and community level, as has previously been shown by Wieczynski et al. (2019) who found opposite trait-climate relationships at the species and community levels. In this study, we found that trait-trait relationships had changed dramatically from the species to community levels, the latter showing stronger correlations, particularly the relationships between economic (excluding SLA) and hydraulic traits (Table 2). A persistent combination of economic and hydraulic traits might be more cost-effective, and Yin et al. (2018) found coupled relationships between leaf economic and hydraulic traits using 47 woody species on the Loess Plateau where considerable drought stress persists. Therefore, our results indicated that weak trait-trait relationships made plant species occupy multifarious niche dimensions, but strong interdependence among community–scale leaf traits resulted in plant communities adopting a cost-effective strategy to improve resource use efficiency and/or resistance to stress (Flores-Moreno et al., 2019).

### Climate Drives the Distributions of Leaf Traits Within Communities

Temperature, precipitation and aridity index influence the distributions and thus diversity of traits within communities. Consistent with ecological theory, certain leaf traits (SLA, N and Chl content, stomatal density and stomatal area fraction) had more even trait distributions in plant communities with lower resource availability (i.e., lower MAT, MAP, AI) (Cornwell and Ackerly, 2009). Further, such evenness of leaf traits were more robustly related to MAP than to MAT. Indeed, previous studies have demonstrated that specific leaf area (SLA), nitrogen content (N), and chlorophyll content (Chl) were significantly influenced by temperature and precipitation (Liu et al., 2017; Li et al., 2018; Zhao et al., 2019). Liu et al. (2018) posit that water availability was the main driver of latitudinal patterns of stomatal density (*d*) and stomatal area fraction (*f*) at the regional scale. Consistent with these findings, the ratio of palisade to spongy thickness was also distributed more evenly at sites with lower water availability, and highlights coordination between traits that scale to optimize resource-use efficiency (Bruelheide et al., 2018). Overall, when water is the main limitation within a community, species diverge in hydraulic traits rather than economic traits that scales to the whole community to use water effectively while promoting the coexistence of multiple species.

Indeed, the distribution of leaf economic traits was more even under lower temperature and precipitation, while the distribution of hydraulic traits was more even only under lower water availability. This indicates that assembly of leaf economic and hydraulic traits in terms of functional evenness would differ depending on the temperature and water availability of a community, which permits communities greater flexibility to adapt to multifarious environments. We boldly speculated that under severe drought stress and suitable temperature, plant communities assembly with species with hydraulic traits more evenly distributed, given their mechanistic link with water use and to maximize resource use efficiency. By contrast, under low temperature and suitable water availability, plant communities assemble with species with economic traits more evenly distributed within the community. Moreover, the independent assembly of multiple trait dimensions within communities improves their adaptability.

### The Assembly of Leaf Functional Traits Within Community Optimized Their Productivity

Establishing the linkages between plant functional traits and ecosystem functioning is a fundamental goal for ecologists. Studies have found that community-aggregated leaf economic traits (Li et al., 2018; Tang et al., 2018), stomatal traits (Wang et al., 2015), and anatomical traits (He et al., 2018) and other plant traits (Diìaz and Cabido, 2001; Lavorel and Garnier, 2002) had a significant influence on ecosystem productivity. Recently, Gross et al. (2017) in drylands and Zhang et al. (2019) in grasslands found that not only community-weighted mean of plant traits, but their variance, skewness, and kurtosis also play important roles in optimizing ecosystem functions. In this study, we found that the influence of leaf economic-hydraulic trait moments (mean, variance, skewness, and kurtosis) on GPP in observed communities were significantly higher than those in the null communities, indicating that assembly of leaf economic and hydraulic traits within community optimized their productivity. Further, the strong coordination between leaf economic and hydraulic traits at the community level allows plant communities to function efficiently and take up resources faster (Flores-Moreno et al., 2019). This would directly affect ecosystem productivity. The independent assembly of multi-dimensional traits in terms of functional evenness, which improved the adaptability of plant communities, also plays an important role in optimizing ecosystem productivity.

Certain potential limitations of our study should be explicitly stated. Although leaf samples were only collected from 4 individual for each species, the species composition was almost completely different among nine typical forests, and interspecific variation of traits was much greater than the intraspecific variation (Reich et al., 1999; Garnier et al., 2001). Therefore, conclusions based on comparison of trait network coordination and distribution within and across communities are robust. Leaf venation traits are also fundamental components of leaf hydraulic traits, and only focusing on stomatal traits might provide limited inference. Further, compared with null communities, the coordination between economic traits and hydraulic traits in observed plant communities, and the explanation of these traits on GPP, were not strongest given that trait multifunctionality was common and any single plant function also depended on multiple traits and their interdependence (Sack and Buckley, 2019). Thus, parameters of observed communities might be far from optimal for community assembly based on single or a limited number of traits. Analyses combining the impacts of many trait dimensions would likely result in observed parameters that reflect more optimal community assembly via optimal trait evolution. This would be reflected in observed values being completely outside the ranges generated by the null models. An extensive suite of functional traits and systemic field investigations are needed to further elucidate the role of multiple traits during community assembly.

## Data Availability Statement

The datasets generated for this study are available on request to the corresponding author.

## Author Contributions

NH conceived the ideas. CL, YL, and JZ collected the data and led the writing of the manuscript. AB revised the manuscript. All authors contributed critically to the drafts and gave the final approval for publication.

## Conflict of Interest

The authors declare that the research was conducted in the absence of any commercial or financial relationships that could be construed as a potential conflict of interest.

## Footnotes

1 http://www.resdc.cn/

2 https://modis.gsfc.nasa.gov/data/dataprod/mod17.php
