# Supplementary material for: Optimal Community Assembly Related to Leaf Economic- Hydraulic-Anatomical Traits
Source: Front Plant Sci. 2020 Mar 25;11:341. doi: 10.3389/fpls.2020.00341 (PMC7109333; doi:10.3389/fpls.2020.00341)
Supplement: Supplementary file 1 [file Data_Sheet_1.docx]

**Optimal community assembly related to leaf economic-hydraulic-anatomical traits**

**Running title:** **Leaf traits and optimal community assembly**

Congcong Liu^1,2^, Ying Li^1,3^, Jiahui Zhang^1,2^, Alec S Baird^4^, Nianpeng He^1,2,5*^

1 Key Laboratory of Ecosystem Network Observation and Modeling, Institute of Geographic Sciences and Natural Resources Research, Chinese Academy of Sciences, Beijing 100101, China

2 College of Resources and Environment, University of Chinese Academy of Sciences, Beijing 100049, China

3 The Key Laboratory for Forest Resources& Ecosystem Processes of Beijing, Beijing Forestry University, Beijing, China

4 Department of Ecology and Evolutionary Biology, University of California Los Angeles, 621 Charles E. Young Drive South, Los Angeles, CA 90095, USA

5 Institute of Grassland Science, Northeast Normal University, and Key Laboratory of Vegetation Ecology, Ministry of Education, Changchun 130024, China

*Correspondence author Nianpeng He (henp@igsnrr.ac.cn).

Tel.: +86-10-64889263

Fax: +86-10-64889399

**Table S1 Location and key properties of nine contrasting forests along the North-South Transect of Eastern China (NSTEC)**

|  | Latitude  (°, N) | Longitude  (°, E) | Altitude  (m) | MAT ^‡^  (°C) | MAP  (mm) | AI | Forest type (No. sampled plant species) |
| --- | --- | --- | --- | --- | --- | --- | --- |
| HZ ^†^ | 51.76 | 123.29 | 850 | -5.3 | 502.1 | 106.8 | Cold temperate coniferous forest (15) |
| LS | 47.18 | 128.89 | 401 | 0.1 | 612.5 | 60.6 | Temperate conifer broad leaf mixed forest (23) |
| CB | 42.40 | 128.09 | 758 | 1.7 | 811.0 | 69.3 | Temperate conifer broad leaf mixed forest (29) |
| DL | 39.97 | 115.48 | 972 | 6.5 | 509.0 | 30.8 | Warm temperate deciduous broad--leaved forest (16) |
| TY | 36.68 | 112.10 | 1668 | 5.7 | 549.7 | 35.0 | Warm temperate deciduous broad-leaved forest (20) |
| SN | 31.32 | 110.49 | 1510 | 9.7 | 1101.1 | 55.9 | North subtropical deciduous evergreen mixed forest (44) |
| JL | 24.57 | 114.44 | 562 | 17.7 | 1718.2 | 62.0 | Subtropical evergreen broad-leaved forest (74) |
| DH | 23.17 | 112.54 | 240 | 21.7 | 1683.1 | 53.1 | South subtropical monsoon evergreen broad-leaved forest (78) |
| JF | 18.74 | 108.86 | 809 | 19.3 | 1407.1 | 48.0 | Tropical monsoon forest (95) |

^†^ HZ, Huzhong; LS, Liangshui; CB, Changbai; DL, Dongling; TY, Taiyue; SN, Shennong; JL, Jiulian; DH, Dinghu; JF, Jianfeng;

^‡^ MAT, mean annual temperature; MAP, mean annual precipitation; AI, de Martonne aridity index.

**Table S2 species-specific equations in this study.**

See Excel

**Table S3 Principal component analysis (PCA) of mean annual temperature (MAT) and precipitation (MAP)**

| Sites | PC1 | PC2 |
| --- | --- | --- |
| HZ ^†^ | -1.73 | -0.37 |
| LS | -1.17 | -0.12 |
| CB | -0.77 | -0.27 |
| DL | -0.83 | 0.52 |
| TY | -0.83 | 0.40 |
| SN | 0.24 | -0.07 |
| JL | 1.72 | -0.33 |
| DH | 1.97 | 0.02 |
| JF | 1.40 | 0.23 |
| % of variance | 94.9 | 5.1 |

**Table S4 Pearson correlation coefficients between climate and the distance (δ) of the observed kurtosis to the boundary kurtosis**

|  | MAT | MAP | AI | PC1_MAT_MAP_ |
| --- | --- | --- | --- | --- |
| δ SLA | 0.75*** | 0.86*** | 0.15 | 0.80*** |
| δ N | 0.61*** | 0.69*** | 0.03 | 0.65*** |
| δ Chl | 0.42* | 0.56** | 0.25 | 0.49** |
| δ *d* | 0.23 | 0.39* | 0.47** | 0.30 |
| δ *s* | 0.59*** | 0.61*** | -0.07 | 0.64*** |
| δ *f* | -0.33 | 0.03 | 0.82*** | -0.16 |
| δ PT | 0.21 | 0.11 | -0.21 | 0.19 |
| δ ST | 0.17 | 0.13 | 0.03 | 0.18 |
| δ PT/ST | 0.16 | 0.37* | 0.58*** | 0.24 |

^†^ SLA, specific leaf area; Chl, leaf chlorophyll content; N, Leaf nitrogen content; *d*, stomatal density; *s*, stomatal size; *f*, stomatal area fraction; PT, palisade tissue thickness; ST, spongy tissue thickness; PT/ST, ratio of PT to ST.

^‡^ δ was log-transformation of the distance between observed kurtosis and boundary kurtosis. Because some original data with very low values close to 0, I added one to all values before log-transformation.

^‡^ MAT, mean annual temperature; MAP, mean annual precipitation; AI, de Martonne aridity index. PC1_MAT_MAP_, PC1 scores of MAT and MAP (first PCA axis accounted for 94.9% of total variation).

*, *p* < 0.05; **, *p* < 0.01; ***, *p* < 0.001.

**Fig.S1 Changes in climate along latitude.**
